# Supplementary material for: The causal relationship between the human gut microbiota and pyogenic arthritis: a Mendelian randomization study
Source: Front Cell Infect Microbiol. 2024 Nov 26;14:1452480. doi: 10.3389/fcimb.2024.1452480 (PMC11629706; doi:10.3389/fcimb.2024.1452480)
Supplement: Supplementary file 1 [file DataSheet1.zip › mendelian test/ebi-a-GCST90027609.csv_leave_one_out.pdf]

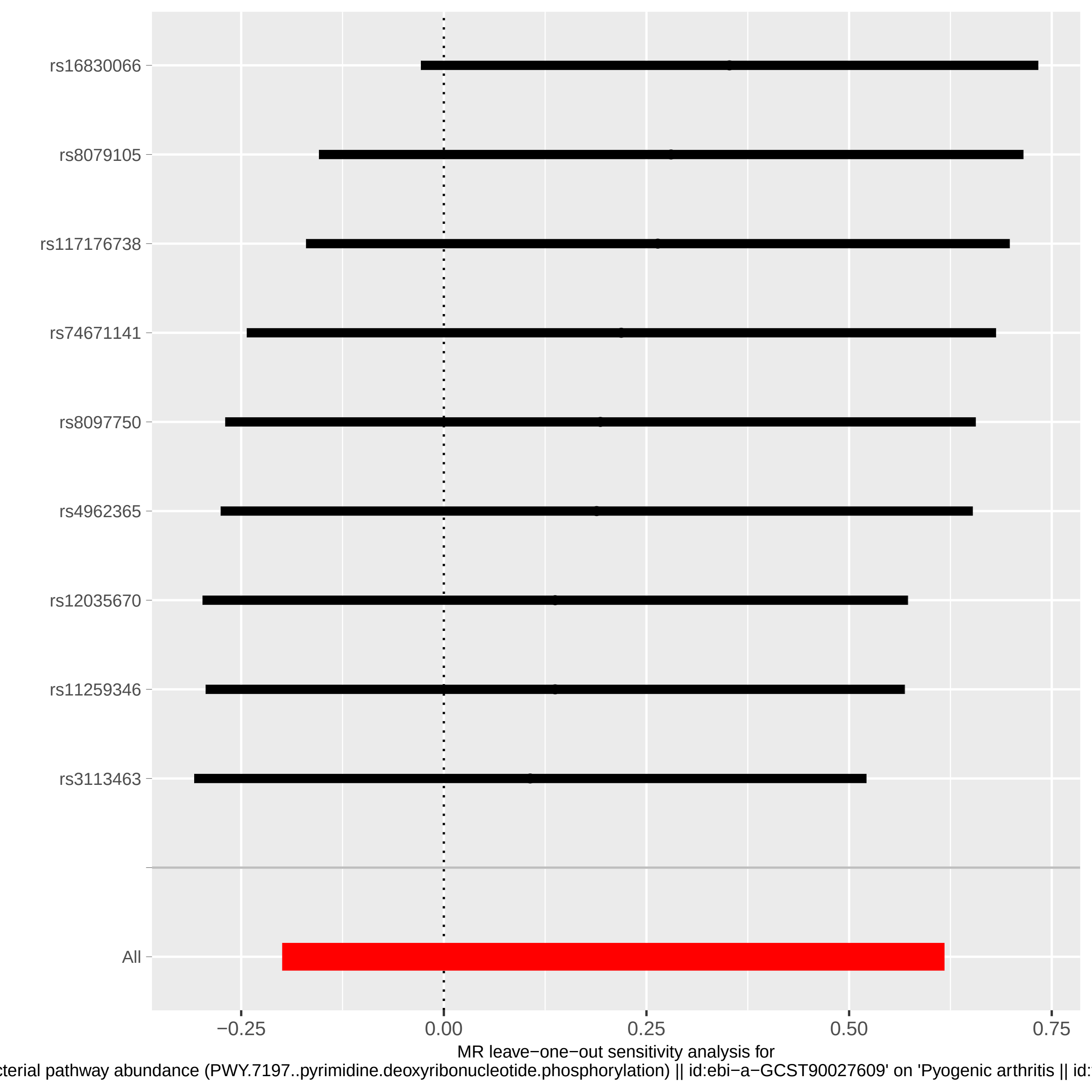

rs16830066
rs8079105
rs117176738
rs74671141
rs8097750
rs4962365
rs12035670
rs11259346
rs3113463
All
−0.25
0.00
0.25
0.50
0.75
MR leave−one−out sensitivity analysis for
cterial pathway abundance (PWY.7197..pyrimidine.deoxyribonucleotide.phosphorylation) || id:ebi−a−GCST90027609' on 'Pyogenic arthritis || id:
